# Functional conservation in genes and pathways linking ageing and immunity

**DOI:** 10.1186/s12979-021-00232-1

**Published:** 2021-05-14

**Authors:** Daniel K. Fabian, Matías Fuentealba, Handan Melike Dönertaş, Linda Partridge, Janet M. Thornton

**Affiliations:** 1 European Molecular Biology Laboratory, European Bioinformatics Institute, Wellcome Genome Campus, Hinxton, UK; 2 Institute of Healthy Ageing, Department of Genetics, Evolution and Environment, University College London, London, UK; 3 Max Planck Institute for Biology of Ageing, Cologne, Germany

**Keywords:** Immunity, Ageing, Lifespan, Longevity, Immunosenescence, Conservation

## Abstract

At first glance, longevity and immunity appear to be different traits that have not much in common except the fact that the immune system promotes survival upon pathogenic infection. Substantial evidence however points to a molecularly intertwined relationship between the immune system and ageing. Although this link is well-known throughout the animal kingdom, its genetic basis is complex and still poorly understood. To address this question, we here provide a compilation of all genes concomitantly known to be involved in immunity and ageing in humans and three well-studied model organisms, the nematode worm *Caenorhabditis elegans*, the fruit fly *Drosophila melanogaster*, and the house mouse *Mus musculus*. By analysing human orthologs among these species, we identified 7 evolutionarily conserved signalling cascades, the insulin/TOR network, three MAPK (ERK, p38, JNK), JAK/STAT, TGF-β, and Nf-κB pathways that act pleiotropically on ageing and immunity. We review current evidence for these pathways linking immunity and lifespan, and their role in the detrimental dysregulation of the immune system with age, known as immunosenescence. We argue that the phenotypic effects of these pathways are often context-dependent and vary, for example, between tissues, sexes, and types of pathogenic infection. Future research therefore needs to explore a higher temporal, spatial and environmental resolution to fully comprehend the connection between ageing and immunity.

**Supplementary Information:**

The online version contains supplementary material available at 10.1186/s12979-021-00232-1.

## Background

Organisms are constantly challenged by various types of pathogens, which inflict numerous negative effects on health and fitness of infected hosts. A speedy resolution of pathogenic infections thus limits their deleterious consequences and is consequently beneficial for survival, providing an adaptive advantage. Hosts achieve immunity through a variety of mechanisms. Foremost, the innate and adaptive immune systems represent the two major routes of promoting resistance to infectious organisms. Immune system activation generally triggers the expression of cytotoxic molecules, such as ***a***nti***m***icrobial ***p***eptides (**AMPs**) or ***r***eactive ***o***xygen ***s***pecies (**ROS**), and the recruitment of specialized immune cells to the infected tissue that inhibit and destroy microbial intruders. In addition, hosts alter their own physiological state upon infection to create an undesirable environment for pathogens. Although these defence mechanisms support survival in the presence of pathogens, maintaining and mounting an immune response can also be associated with two types of costs. First, physiological trade-offs between immunity and other metabolically expensive traits, in particular growth and reproduction, have been identified across the tree of life, including plants [1], insects [2], and vertebrates [3]. Second, because immune mechanisms are not entirely specific to pathogens, they cause adverse side-effects, for instance, by harming the host tissue. Organisms thus need to find a delicate balance between allocation of limited resources and the intensity of the immune response to optimize their evolutionary fitness.

**Fig. 1:**
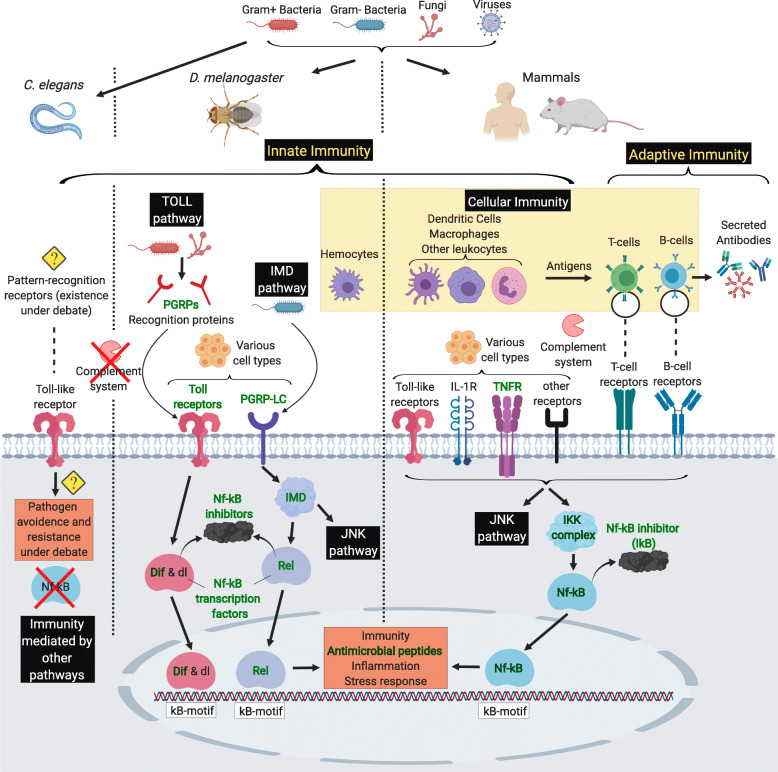
Major differences in immunity of *C. elegans*, *D. melanogaster* and mammals. Invertebrates, such as nematodes and insects, represented by *C. elegans* and *D. melanogaster* respectively, completely rely on innate immunity upon infection, while vertebrates (represented by mammals) have also evolved an adaptive immune system. Cellular immunity occurs in fruit flies as hemocytes (immune cells in the hemolymph), and as different types of leukocytes in mammals, but is absent in worms. In contrast to this, the germ-line encoded complement system is unique to mammals. Moreover, pattern recognition receptors (PRRs) of the innate immune system – most prominently the Toll- and Toll-like receptors, are incremental for pathogen-recognition in fruit flies and mammals, but whether the single Toll-like receptor homolog in *C. elegans* fulfils the same function is still under debate. Finally, Nf-κB transcription factors, which regulate the expression of immune-related genes, are central to *D. melanogaster* and mammalian immunity, but have not been identified in *C. elegans*. The well-known cross-talk between immune and JNK MAPK signalling is indicated, but notably several other pathway interactions do also exist. Immunity genes and protein complexes also associated with lifespan and ageing are marked in green and bold

The adverse properties of immunity become particularly apparent as organisms age. The immune system and the inflammatory response are subject to strong age-dependent loss of homeostasis, resulting in age-related immunopathology known as immunosenescence – a term first coined by Roy Walford in the 1960s [4]. The decline in normal immune function manifests in a multitude of effects detrimental for health and longevity, including increased susceptibility to pathogens, decreased vaccine-response, chronic inflammation or ‘inflammaging’, impaired wound healing, and a higher incidence of cancer [5, 6]. Age-associated increases in auto-immunity factors, such as self-reacting auto-antibodies, are also common but do not necessarily cause a higher incidence of auto-immune diseases in elderly, potentially due to enhanced protective mechanisms which occur in parallel [7]. Interestingly, almost all species including vertebrates and invertebrates are subject to immunosenescence, even though they vary vastly in their immune systems, life history, lifespans, and ecological niches. Figure 1 summarises the components and pathways involved in the immune system of the four different organisms. It is well known that survival upon pathogen exposure declines with age at infection in the nematode worm *Caenorhabditis elegans* [8, 9], the fruit fly *Drosophila melanogaster* [10, 11], and several mammals [12–14], demonstrating that immunosenescence affects both the innate and adaptive immune system. Counterintuitively, the reduced pathogen resistance is accompanied by an increase in basal expression levels of several innate immunity and inflammatory genes, such as antimicrobial peptides, cytokines, and complement system factors in flies [15, 16], mice [17, 18], and humans [6, 19], although this has not been reported in *C. elegans* to our knowledge. Thus, even though baseline immunity is higher in older individuals, realized immunity, in terms of pathogen defence, depreciates. Moreover, this ramping up of baseline immunity with age is thought to be the main reason for the deleterious, pro-inflammatory state of inflammaging [5, 6]. In parallel to this, innate immunity cells decline in phagocytic ability with age in *D. melanogaster* [20] and mammals [6], while changes in the adaptive immune system are characterized by a depletion in naïve cells and a rise in memory cells [5, 6], theoretically making the organism less adaptable to novel antigens. The exhaustion in naïve immune cells with age makes the host reliant on memory cells, which are specific to pathogens encountered in the past, while defence against novel antigens is weakened. This depletion is thought to be driven by two major factors: (1) the genetically programmed decline in mass of the thymus where T-cells mature, known as thymic involution, and (2) life-long accumulated antigenic exposure diminishing naïve immune cells [5, 6].

It has also become apparent that many age-associated diseases, such as Alzheimer’s, Parkinson’s, diabetes, cancer, and atherosclerosis, exhibit a significant immunological and inflammatory component [21–24], as further corroborated by studies in model organisms (e.g. [25–27]). Identifying the interlinked genes and pathways in different species, which contribute pleiotropically to phenotypes associated with ageing, lifespan and immunity, could help to identify common molecular mechanisms of the immunity-ageing cross-talk.

In this review, we utilise public databases and inhouse manual curation to assemble a resource of genes known to affect both immunity and ageing (from here on called ‘immuno-ageing’ factors) in two invertebrates, *C. elegans* and *D. melanogaster*, and two mammalian species, the house mouse (*Mus musculus*) and humans (*Homo sapiens*). We then identify conserved genes between these species based on human orthologs and associate them to core immuno-ageing pathways, which we discuss in this review. We finish by outlining open questions and necessary experiments to further understand the complex relationship between ageing and immunity.

## Evolutionarily conserved immuno-ageing factors

**Fig. 2:**
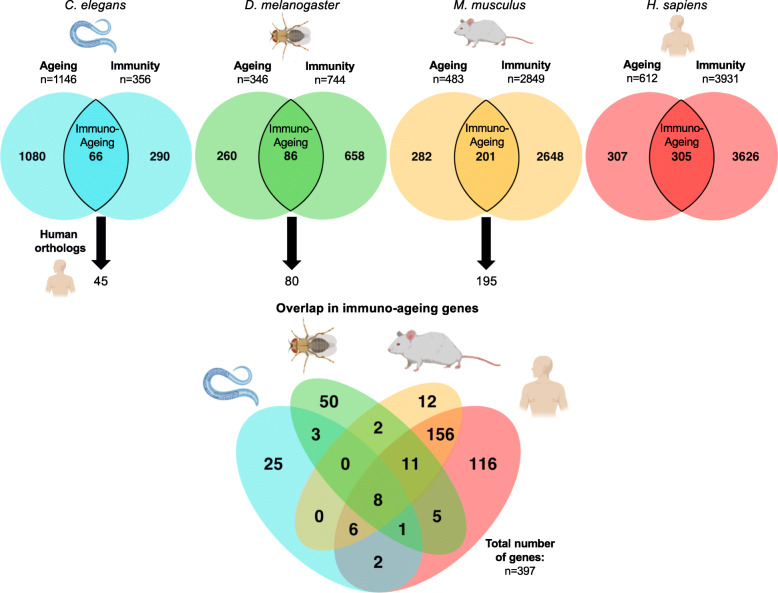
Overlap in 437 Human immuno-ageing orthologs between *C. elegans* (blue), *D. melanogaster* (green), *M. musculus* (orange), and *H. sapiens* (red). Orthologs were obtained using DIOPT. Details on shared orthologs, original gene names and methods are given in Table S2

To classify pathways likely mediating the cross-talk between ageing and immunity, we first combined all genes within the ***G***ene ***O***ntology (**GO**) and KEGG terms related to immunity or ageing, and further included annotations from two ageing (GenAge [28], AgeFactDB [29]) and three immunity databases (insect innate immunity database IIIDB [30], InnateDB [31], immunome knowledge base IKB [32]), resulting in an ‘ageing’ and ‘immunity’ gene list for each species. The definition of ageing and immunity genes varies among these databases, but broadly, the function of the genes in our lists are derived from experimental evidence and computational inference. Next, we identified pleiotropic immuno-ageing genes by intersecting the ageing and immunity gene lists of each species (Fig. 2). Details on gene list construction, number of genes per database, and lists of identified ageing, immunity and immuno-ageing genes across the four species are available in Table S1. We obtained different numbers of ageing, immunity and immuno-ageing genes across the four species, which could be driven by biological differences, such as varying number of genes modulating ageing and immunity between the species or the total number of genes in each species. From a technical point of view, two major caveats of our approach, that also influence these differences, are study-preferences for either ageing or immune function in a particular model, and researcher-biases in studying specific genes and pathways considered more important, which has previously been shown to influence the analyses of ageing-related genes [33].

**Fig. 3:**
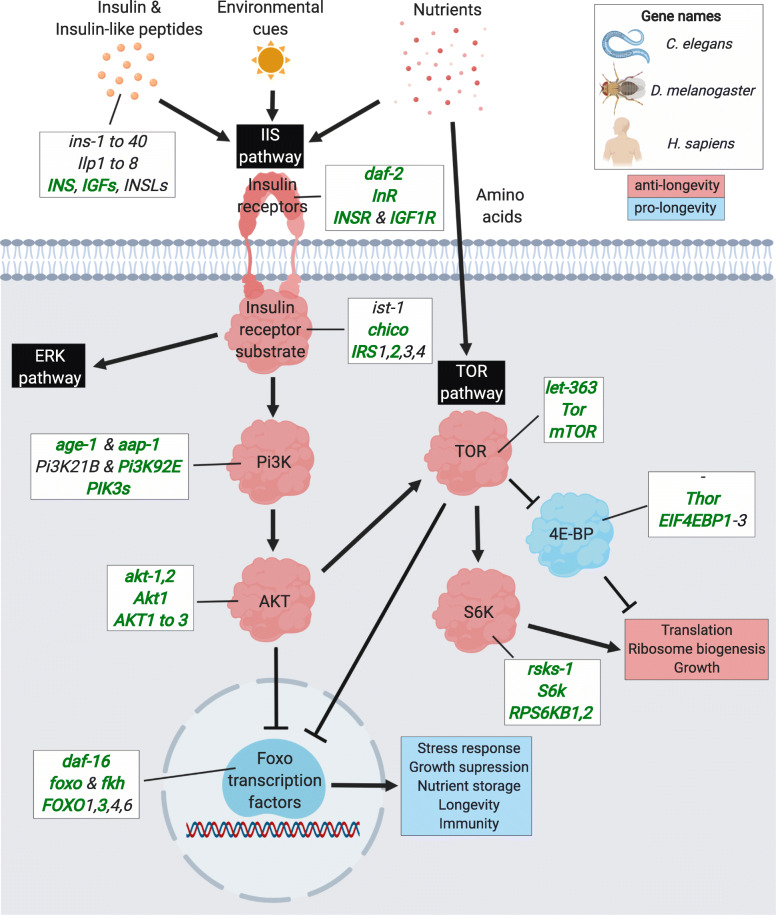
Simplified overview of the insulin-insulin-like growth factor and TOR signalling (IIS/TOR) network. Activation of IIS/TOR network signalling is induced by insulin and insulin-like peptides, environmental cues and nutrients, and leads to a signalling cascade promoting growth and reducing lifespan. In absence of IIS/TOR signalling, such as under dietary restriction, Foxo transcription factors and the 4E-BP inhibitor of translation inhibit growth and promote longevity. Factors stimulating IIS/TOR signalling and thought to reduce lifespan are shown in red, those generally beneficial for lifespan in blue. Gene names are given in the white boxes in the order of *C. elegans*, *D. melanogaster* and *H. sapiens* (overall, equal to *M. musculus*). Genes with pleiotropic effects on immunity are marked in green and bold

**Fig. 4:**
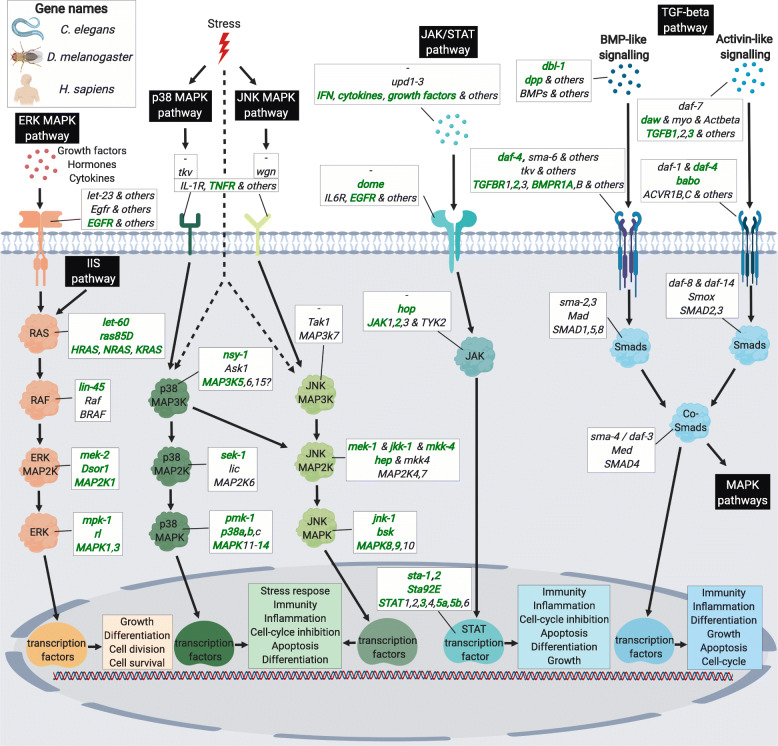
MAPK, JAK/STAT and TGF-β pathways regulate ageing and immunity. Multiple signalling cascades in addition to the IIS and TOR pathways contribute to ageing, longevity and immunity. Three MAPK pathways are conserved in worms, flies and mammals. The ERK-MAPK pathway is activated by growth and other factors through binding to receptors, including *EGFR*, and can further be triggered by the IIS pathway. Activity leads to transcription factors mainly promoting growth, cell division and differentiation of cells. In contrast, p38 and JNK MAPK pathways are classically considered to respond to stress including infections, while the JNK cascade is also mediated by Nf-κB signalling (Fig. 1). The multiple transcription factors downstream of p38 and JNK stimulate inflammation, immunity, longevity and other functions. JAK/STAT signalling is triggered by *upd* cytokines in *D. melanogaster*, and interferons, multiple cytokines and other factors in mammals. Upon receptor binding, the JAK/STAT signalling cascade leads to activation of STAT transcription factors, which are best known for fostering inflammation and immunity, particularly against viral infections. Notably, JAK/STAT in *C. elegans* only includes the two STAT transcription factors, *sta-1* and *sta-2*, but does not share the same upstream factors as fruit flies and mammals. Finally, the branches of TGF-β pathways are activated by BMPs, activins and other factors. The signalling cascade regulates multiple transcription factors, leading to immunosuppression, anti-inflammatory responses and several other physiological effects. Notably, the pathways show various amounts of cross-regulation among each other but also with the IIS/TOR network and the Nf-κB cascade, and for simplicity, only well-known cross-talk is indicated. Gene names are given in the white boxes in the order of *C. elegans*, *D. melanogaster* and *H. sapiens* (overall, equal to *M. musculus*). Genes and factors concomitantly involved in lifespan, ageing, and immunity are marked in green and bold

Next, we asked if there are any evolutionarily conserved immuno-ageing genes, which might be interesting candidates for modulating ageing, lifespan and immunity across evolutionarily distant species, and identified in total 437 orthologs using DIOPT [34] based on human genes (Fig. 2 and Table S2). To further identify the most conserved genes, we overlapped orthologs across the four species. As expected, the overlap between mammals and invertebrates is low, reflecting in part the lack of adaptive immunity in flies and worms. Only ten highly conserved immuno-ageing genes were common in all four species. Of these, the six genes *akt-1/Akt1/AKT2*, *age-1/Pi3K92E/PIK3CD*, *daf-2/InR/IGF1R*, *daf-16/foxo/FOXO3*, *let-363/Tor/MTOR*, and *rsks-1/S6k/RPS6KB2* (order of gene names: *C. elegans* / *D. melanogaster* / mammals) are members of the ***i*nsulin/*i*nsulin-like growth factor** ***s*ignalling** (IIS) and the ***t*arget-*o*f-*r*apamycin** (TOR) pathways and are well-known for their conserved roles in ageing (Fig. 3). The other four genes in this category act within the ERK and p38 MAPK pathways and include the *mpk-1/rl/MAPK1*, *pmk-1/p38a and b/MAPK14*, *mek-2/Dsor1/MAP2K1,* and *let-60/Ras85D/HRAS* (Fig. 4). While the ERK MAPK pathway mainly gets activated through IIS and growth factors and promotes proliferation, the p38 MAPK pathway is mostly known for its response to environmental stresses [35]. Inspection of triple and double overlaps between invertebrates and mammals included further IIS/TOR network components, as well as genes related to the JNK MAPK pathway (Fig. 4), which performs similar downstream functions as the p38 cascade [35]. Moreover, we found genes related to the immuno-supporting JAK/STAT [36, 37], and the TGF-β pathway, which is thought to have a great diversity of context-dependent roles in cellular and physiological function, including immunity [38, 39] (Fig. 4). In addition, the Nf-κB signalling components were shared between fruit flies and mammals, since this pathway is thought to be absent in *C. elegans* (Fig. 1). In general, the overlaps between invertebrates and mammals comprised genes acting broadly across the signalling transduction, such as ligands, receptors and transcription factors. We also found some noteworthy genes partially controlled by Foxo transcription factors, including *sod-3/Sod2/SOD2* and *ctl-1/Cat/CAT* which are involved in the clearance of oxidative stress, *atm-1/tefu/ATM* that functions in DNA repair and telomere maintenance, and the antiviral gene *dcr-1/Dcr-2/DICER1* (see discussion below).

In summary, our analysis suggests that the IIS/TOR network together with ERK/p38/JNK MAPK, JAK/STAT, TGF-β, and the Nf-κB pathways have highly conserved mechanisms in immunity, lifespan and ageing. In the remainder of the review, we shall explore the roles of each of these pathways on the immunity-lifespan cross-talk and immunosenescence as revealed in studies on the different organisms.

## The IIS/TOR network is a key regulator of ageing and immunity

### Invertebrate IIS/TOR network

The nutrient-sensing IIS/TOR signalling network is among the best understood molecular determinants of ageing and lifespan. Reducing the activity of IIS and/or TOR signalling through genetic or nutritional interventions (e.g. dietary restriction) has long been established to prolong lifespan in yeast, nematodes, insects, and mammals [40–42].

Pioneered by studies in *C. elegans*, a major role of the IIS/TOR network in immunity has been demonstrated in addition to regulating lifespan. A seminal discovery establishing this connection is that long-lived loss-of-function mutants of *daf-2*, encoding the ***i***nsulin-like ***g***rowth ***f***actor 1 (IGF-1) receptor, are more resistant to gram-positive and gram-negative bacteria [43–45] (Fig. 3**)**. These results are further supported by the observation that bacterial infection of the pharynx, thought to be a major cause of early death in *C. elegans* [46], was less severe in *daf-2* loss-of-function mutants during ageing than in wildtype worms [47]. Increased pathogen resistance has also been shown for ***p***hosphatidyl-***i***nositol ***3 k***inase (PI3K) AGE-1 null mutants [43, 45, 48], while the downstream forkhead transcription factor DAF-16, which is suppressed upon IIS activation, is usually conferring pathogen resistance [43, 45, 49, 50].

Comparable to worms, downregulating insulin signalling through loss-of-function of the insulin receptor substrate gene *chico* in *D. melanogaster* (Fig. 3) is beneficial for longevity and increases resistance to pathogenic bacteria, although effects vary dependent on the pathogens or the *chico* null allele used [51–53]. Surprisingly, AMP expression in *chico* mutants is generally decreased or equal to that in wildtype flies, which represents a poorly understood mismatch between potential and realized immunity [52, 53]. Moreover, similar to worms, lowered *foxo* expression has negative consequences on survival upon infection potentially because of a concurrent downregulation in AMPs [54–56].

The immunity modulating features further extend to the TOR pathway. Reduced TOR pathway activity has been associated with improved lifespan and enhanced immunity by promoting autophagy in *C. elegans* [57–59] and inhibiting protein translation in *D. melanogaster* [60, 61]. However, the effects of TOR in fruit flies are not always clear-cut. While induction of AMP expression independent of the Nf-κB pathway and improved phagocytic ability of hemocytes have been observed upon lowered TOR pathway activity in *D. melanogaster*, it may deter bacterial clearance at the same time [62, 63].

**Fig. 5:**
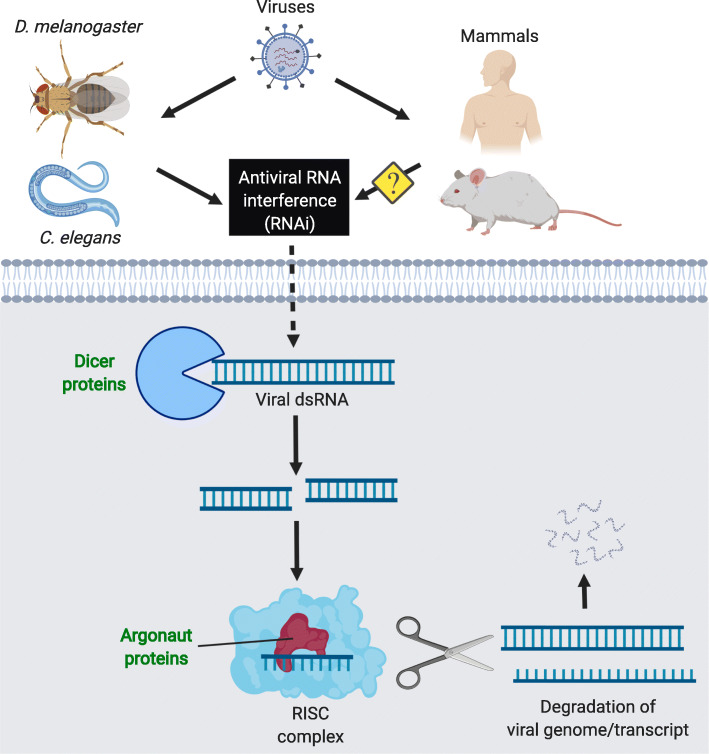
Antiviral RNAi pathway. The antiviral RNA interference (RNAi) pathway shows genetic and functional conservation across *D. melanogaster*, *C. elegans* and mammals. In both, *C. elegans* and *D. melanogaster*, antiviral RNAi is thought to be a major innate defence against viruses. However, even though antiviral RNAi exists in mammals, its importance is currently under debate as they contain other potent mechanisms of virus defence, such as the interferon response and adaptive immunity. Components also involved in lifespan in at least one of the represented species are marked in green and bold

The similar links between IIS/TOR signalling and pathogen defence in *D. melanogaster* and *C. elegans* might even extend to immunity against viruses. In invertebrates, the RNAi pathway confers immunity against viruses, and in worms further contributes to bacterial infection [64–68] (Fig. 5). Several studies suggest that in *C. elegans*, reduced activity of insulin signalling causes enhancement of RNAi-mediated immunity, including transposable element silencing [69–71]**,** which is also thought to be beneficial for longevity in fruit flies and mammals likely due to their neurodegenerative and inflammatory properties [72–76]. In parallel, it has been shown in arthropods that IIS/TOR activation acts pro-viral, whereas FOXO activity positively regulates the expression of antiviral RNAi genes, thereby increasing survival upon viral infection [77, 78]. Reflecting these findings, inhibition of multiple proteins of the IIS/TOR network was implicated in viral replication in mammals [79–81], but the role of RNAi in mammalian immunity has been controversial [82, 83]. Further supporting a pro-immunity and pro-longevity role of the RNAi pathway, RNAi loss-of-function mutants have a lower lifespan in *C. elegans* [84] and mice [85], and reduced lifespan, stress resistance and antiviral immunity in *D. melanogaster* [75, 86, 87]. In turn, a ubiquitously active RNAi pathway can have detrimental effects on lifespan [88], so that deviations from its wildtype homeostasis may be generally deleterious for ageing.

Altogether, evidence so far converges on the notion that downregulation of the IIS/TOR network has not only positive effects on lifespan but also improves immune function in addition to its metabolic roles. Recent research in Lepidoptera species and mosquitoes shows that the connection between the IIS/TOR network and immunity is not confined to well-established model organisms [89–91]. Moreover, while the three main innate immunity pathways in fruit flies (Toll, IMD, and the antiviral RNAi pathway) are thought to confer resistance to certain types of pathogens, immunity through FOXO regulated genes appears to be general. The effect of the IIS/TOR network on lifespan, however, appears to be slightly more consistent throughout the literature than that for immunity, suggesting that the link might not be omnipresent. Improving lifespan through IIS/TOR may frequently, but not always, cause improved immunity rather than the other way round. Future studies analysing the effect of the IIS/TOR network on lifespan and immunity together in controlled experiments would be crucial to further disentangle the ‘cause-effect’ relationship between both traits.

### Mammalian IIS/TOR network

Are the striking similarities of the IIS-TOR-immunity interplay found in *C. elegans* and insects found in organisms with an adaptive immune system? In parallel to invertebrates, reductions in growth hormone and the insulin signalling pathway were associated with enhanced lifespan and slowed immunosenescence through maintaining young T-cell profiles [92, 93] and decreasing memory relative to naïve T-cells [94, 95]. Indeed, mammalian Foxo transcription factors downstream of IIS/TOR are likely among the key components driving the immuno-ageing cross-talk. Analogous to *C. elegans* and *D. melanogaster*, one of the mammalian Foxo homologs *Foxo3* is a pro-longevity factor, yet is required for survival upon chronic pathogenic infections, expression of antimicrobial peptides, and likely acts anti-inflammatory [96–98]. Although FOXO transcription factors are important mediators of ageing and immunity in mammals, their effects may vary dependent on the FOXO gene and cell type [99–101].

Similarly, TOR signalling is found to be necessary for the innate immune response in mammals, where the functions of the two conserved TOR protein complexes have been extensively studied. In agreement with observations in invertebrate models, inhibiting the mTORC2 complex leads to improved survival upon infection and facilitates expression of pro-inflammatory cytokines mediated by FOXO1 [102, 103]. In contrast, mTORC1 activity was linked to the expression of pro-inflammatory cytokines and type I IFN-γ proteins, which are essential molecules in pathogen resistance [104, 105]. Remarkably, opposing findings in *D. melanogaster*, knockout of two (of three) mammalian inhibitors of translation 4E-BP1 and 4E-BP2, which are inactivated by mTORC1 signalling, increased resistance to viruses by fostering cytokine production [81]. The fact that mTORC1 promotes innate immunity in mammals perhaps explains these contradictory results. Furthermore, there is a large body of work demonstrating the necessity of the TOR pathway in adaptive immunity, where it contributes to antigen presentation, immune cell activation, differentiation, and memory formation (as reviewed by [106]). For example, treating mice with the immunosuppressant drug rapamycin, which only inhibits the mTORC1 complex [107], increased their lifespan and the regenerative ability of hematopoietic stem cells, leading to higher numbers of B-lymphocytes and better survival upon Influenza infection in old individuals [108]. In agreement with this, mTOR inhibition in elderly humans enhanced influenza antibody titres post vaccination, which was concurrent with a reduction in PD-1-positive T-cells that are known to accumulate with age [109]. The fundamental links between the IIS/TOR pathway and immunosenescence are further supported by a tremendous amount of evidence that insulin-like peptide mediated signalling, PI3K activation, and several associated genes function not only in regulation of metabolism and ageing, but are also necessary for innate and adaptive immunity [110–114].

Due to the complexity of the mammalian IIS/TOR network, it is more problematic to generalize its exact effects on immunity and lifespan compared to invertebrates. This is nicely delineated by the different immunological functions of the two mTOR complexes explained above, but also encompasses other components, as demonstrated by the fact that FOXO1 and FOXO3 can have opposing properties supporting survival and apoptosis of T-cells, respectively [99]. A likely explanation for this complexity is repeated gene duplication patterns followed by functional divergence, the fast evolutionary rates of some IIS/TOR genes, and diversifying natural selection [115–117]. An alternative explanation might be that *C. elegans* and *D. melanogaster* were not studied as extensively with regards to the function of IIS/TOR on ageing and immunity in different tissues and treatments. Hypothetically, invertebrates might exhibit a similar functional complexity as mammals do, but it has not yet been detected. Besides nematodes, insects, mice and humans, not many other species have been investigated for an immuno-ageing interplay, although recent studies in fish revealed a cross-talk between immunity and metabolism through insulin-like peptide hormones [118, 119].

## MAPK pathways variably affect ageing and immunity

### Invertebrate ERK MAPK pathways

The ERK MAPK pathway is, like the IIS/TOR network, a central regulator of proliferation and cellular processes (Fig. 4). Thus, its activation might comparably mediate resource allocation to growth at the cost of lifespan and immunity. In contrast to this expectation, the ERK-MAPK pathway appears to promote lifespan and immunity in worms. It has been demonstrated that loss of ERK-MAPK activity resulted in reduced lifespan of *C. elegans* [120], while several genes along the ERK MAPK cascade were shown to promote defence against a bacterial pathogen [121–124]. Interestingly, this suggests that, in the presence of nutrients, activation of the ERK MAPK pathway in *C. elegans* antagonizes the detrimental effects of IIS/TOR signalling on lifespan and immunity.

Opposing findings in worms, ERK MAPK activation demonstrably reduces lifespan in fruit flies and leads to inhibition of the IMD/Nf-κB immunity pathway with reduced survival upon bacterial infection as a consequence [125, 126]. Nevertheless, the regulation of immunity conferred by this pathway could depend on the pathogen. Additional reports indicate that ERK MAPK activation restricts replication of viruses after oral infection in *D. melanogaster*, and this function could be conserved in *Aedes* mosquitos [127, 128].

### Invertebrate p38 and JNK MAPK pathways

*C. elegans* further relies on the p38 MAPK cascade to mount an antimicrobial peptide response and for a functional immune system (Fig. 4) [120, 129, 130]. Similar to the ERK MAPK pathway, activation of the p38 MAPK signalling has positive effects on both pathogen resistance and lifespan in worms [120, 122, 124, 131–135]. However, its impact on lifespan is possibly context-dependent, as demonstrated by studies reporting that p38 MAPK signalling does either not affect lifespan [120, 130] or reduces it, possibly dependent on temperature or the mutant background [132, 136]. Supporting a complicated role in *C. elegans* lifespan, the p38 MAPK pathway is further required for lifespan extension by dietary restriction and reduced insulin signalling, while both of these treatments simultaneously reduce its activity [137].

Same as in *C. elegans*, the p38 MAPK pathway in *D. melanogaster* is involved in stress response and might be required for downregulation of immunity gene expression after infections [138]. In support of its role in immunity, p38 MAPK is important for resistance against bacteria, fungi, and DNA viruses, and further contributes to regulation of the immune system [139–141]. Importantly, p38 MAPK also controls fat and glycogen metabolism in the fat body, which is functionally similar to the mammalian liver, but also AMP expression under bacterial infection, thereby illustrating a case of immune-metabolic cross-regulation [142]. Still, how this pathway affects lifespan is not fully resolved, although recent results suggest a pro-longevity effect in muscle cells, while no effects were observed in neurons [143, 144]. In general, the trend implies similar pro-longevity and pro-immunity roles of the p38 MAPK pathway in *C. elegans* and *D. melanogaster*.

Analogously, the JNK-MAPK signalling pathway, known to stimulate stress resistance (Fig. 4), also promotes longevity and survival to bacterial infections in *C. elegans*, and this is at least partly caused by cross-talk with IIS/TOR and activation of DAF-16 [145–147]. This, however, only partly extends to fruit flies, where JNK activation generally extends lifespan although possibly dependent on the tissue [148–150]. Moreover, JNK is cross-talking with IMD and is required for the activation of antimicrobial peptides [151]. Despite this, reduced JNK signalling activity has been shown to increase survival upon *P. entomophila* infection [152]. This is akin of recent findings in *Anopheles stephensi* mosquitos, where reduced JNK activity led to elevated *Plasmodium* resistance [153].

### Mammalian MAPK pathways

Mammals resemble the MAPK-driven regulation of immunity and ageing observed in *C. elegans* and *D. melanogaster*. Parallel to findings in fruit flies, knockout of the ERK pathway component gene *RasGfr1* in mice had beneficial effects on lifespan, suggesting that ERK activation is limiting lifespan in mammals [154]. In line with this, long-lived dwarf mouse models have reductions in ERK and p38 MAPK signalling along with alleviated immunosenescence [92, 155, 156]. Indeed, p38 MAPK signalling can promote ageing of gut stem cells after activation through mTORC1 [157]. Yet, cardiac-specific knockout of p38⍺ (*Mpk14*) MAPK [158] and two MAPK pathway activators did not impact lifespan in mice [159], suggesting that other functionally redundant genes can compensate for the loss-of-function in this kind of studies.

Mammalian MAPK pathways are further not just involved in innate immunity but also contribute to T- and B-cell survival and activation, as well as inflammation and Nf-κB mediated transcription [160–162]. Yet, their impact on immunity is complex, as demonstrated by the negative effects of JNK1 (*Mpk8*) activation on survival upon fungal infection in mice, which was explained by a reduced production of nitric oxide defence molecules [163].

Similarly, inhibition of the p38 MAPK pathway increased the survival time of influenza infected mice [164] and comparably improved immunity to varicella zoster virus in the human skin [165], likely through reducing pro-inflammatory cytokine production and preventing an overreaction of the immune system also known as a ‘cytokine storm’. Consistent with these results, the ability to resolve inflammation is reduced by increasing p38 MAPK activity in mononuclear phagocytes of elderly humans, but can be restored upon pharmacological inhibition of p38 [166]. Moreover, deficiencies in DUSP genes, that inhibit MAPK pathways in a negative feedback loop, result in deregulation of the inflammatory response (as discussed by Arthur & Ley [167]).

## Context-dependent effects of the JAK/STAT pathway on ageing and immunity

A further conserved immuno-ageing pathway is the JAK/STAT signalling cascade, which regulates numerous cellular processes, but is also central to immunity and the expression of cytokines [36, 168] (Fig. 4). Despite *C. elegans* lacking a homolog of JAK, it retains two functional STAT orthologs that play a role in the defence response to viruses and fungi and reduce lifespan upon knockout [168, 169]. Interestingly, the worm STAT orthologs possibly have opposing roles in immunity, and either promote infection response genes, such as AMPs, dependent on the p38 MAPK and TGF-β pathways [168] or repress them [169].

Comparable to *C. elegans*, JAK/STAT signalling regulates the expression of genes fighting pathogenic infections in *D. melanogaster*, with the difference that the pathway is activated by cytokine-like proteins [37]. Several lines of evidence suggest that activating JAK/STAT signalling is detrimental for lifespan, and the effect size might depend on diet and tissue [170–172]. This implies that JAK/STAT signalling possibly represents a pathway mediating a trade-off between immunity and lifespan, which is akin to the opposite effects of that STAT ortholog *sta-1* on lifespan and immunity in *C. elegans* [169].

Further supporting a conserved role of JAK/STAT in immunity and ageing, loss-of-activity of JAK/STAT genes in mice has negative consequences on immunity, and causes defects in interferon signalling and increased susceptibility to viral infection [173]. In line with a trade-off between immunity and lifespan, administration of a JAK pathway inhibitor reduced inflammation and alleviated cellular senescence [174]. In addition, loss of expression of the JAK/STAT inhibitor *Socs2* was associated with decreased lifespan in mice [175]. Opposite to this, the downstream transcription factor STAT3 was found to be protective against inflammation-induced heart damage, highlighting context-dependency of the immuno-ageing properties of JAK/STAT [176].

## TGF-β signalling affects ageing and immunity dependent on context

The conserved TGF-β pathway acts as a key regulator of physiological homeostasis, as it controls a huge number of cellular functions including growth, differentiation, and apoptosis, often in a context-specific manner [177] (Fig. 4). Perhaps unsurprisingly, considering its diverse roles, it also helps to mediate the relationship between lifespan, ageing and immunity. In *C. elegans*, loss-of-function of the TGF-β homolog *dbl-1* has negative effects on survival upon infection and longevity [178], suggesting positive effects on immunity and ageing. In contrast, TGF-β signalling activation in *D. melanogaster* is associated with a negative regulation of the immune response mediated by the infection/wounding-regulated genes *dpp* and *daw* [179]. These results might again be context-specific: upon infection with pathogenic nematodes, both of these genes were thought to promote survival, but the exact factors involved might vary between adults and larvae [180, 181]. While *dpp* and its downstream transcription factor *Mad* (BMP signalling) are considered to have anti-ageing functions, lifespan effects for *daw*, its receptor *babo*, and downstream factor Smox (Activin signalling) are variable [182, 183]. Nevertheless, TGF-β signalling might help maintaining protein homeostasis, suggested to be a central hallmark of ageing [184].

In mammals, TGF-β signalling is also known to activate MAPK pathways and is similarly crucial for regulating a vast amount of cellular processes, including survival and immunity [38, 39]. Apart of its multifaceted biological roles, TGF-β knockout mice exhibited higher inflammation, auto-immunity and reduced lifespan, and the detrimental effects were alleviated by further removing MHC class I functionality necessary for CD8+ T cell development [185]. As before, context-dependency of TGF-β exists because lifespan was reduced in females, but not in males in mice with impaired TGF-β in salivary glands [186].

## Nf-κB signalling mediates a trade-off between immunity and ageing

While the immuno-ageing pathways outlined above are conserved between *D. melanogaster* and *C. elegans*, central parts of fruit fly and mammalian immunity signalling missing in worms are Nf-κB transcription factors (Fig. 1). Nf-κB signalling has central functions in innate and adaptive immunity, and is triggered upon activation of several immune receptors, most prominently Toll-like receptors, during pathogenic infection. Downstream, it results in the expression of pro-inflammatory genes, antimicrobial peptides, and regulates the activation, differentiation and survival of immunity cells [187]. However, Nf-κB signalling having an influential role in ageing and lifespan is less established. In *Drosophila*, overexpression of two immuno-stimulating pattern recognition receptors, the intracellular PGRP-LE and the transmembrane PGRP-LC, both upstream of Toll and IMD confer an inflammatory state with detrimental effects on lifespan [188, 189]. In line with this, genetic manipulation of immune-suppressive PGRP genes implies that activation of the immune system is unfavourable for longevity [190, 191]. As expected, the activity of IMD and Toll pathway is overall detrimental for lifespan, however, might also partially depend on tissue, sex, the gut microbiome, and type of genetic intervention [26, 192–204]. The effect on lifespan might be dependent on the expression of downstream AMPs, which can take both pro- and anti-longevity roles, with some study-specificity regarding their effect on lifespan [193, 196, 204]. As such, the increased immune system activity associated with ageing is perhaps harmful for lifespan because detrimental components outweigh beneficial factors.

One emerging mechanism explaining lifespan regulation by immune signalling pathways in *Drosophila* is through cross-talk with the IIS/TOR network. Innate immune signalling has been found to reduce insulin and TOR pathway activity, where this might be partially directed by cross-talk with the JNK pathway [148, 149, 194, 205–208]. In contrast to this, IMD can also activate insulin signalling in enteroendocrine cells, and plays a role in lipid metabolism and development, therefore highlighting tissue-specific effects and the difficulty to formulate general system-wide conclusions [209]. These findings pose a challenging molecular conundrum: although Nf-κB inhibits the IIS/TOR signalling network in certain conditions, which usually results in increased lifespan, it concomitantly has negative effects on lifespan.

Equivalent to findings in *Drosophila*, there is increasing evidence that in mammals, Nf-κB signalling stimulates pro-inflammatory responses at the cost of reduced lifespan [210, 211] and has been associated with several age-related pathologies [212, 213]. Indeed, long-lived dwarf mice have decreased levels of inflammatory markers in neurological and other tissues, possibly attributable to lowered JNK and Nf-κB activity, further confirming that these mice suffer less from chronic inflammation with age [156, 214]. Thus, while Nf-κB activity is required for pathogen resistance and inflammation, it is generally detrimental for lifespan in both fruit flies and mammals, representing a conserved trade-off between immunity and ageing.

## Conclusions

In this review, we combined curation and analysis of orthologs between *D. melanogaster*, *C. elegans*, mice and humans to reveal that genes currently known to be pleiotropically involved in immunity, lifespan, and ageing reside in a few core pathways mediating the immuno-ageing interplay (Fig. 2, Table S1, Table S2). Remarkably, several of the most conserved immuno-ageing pathways we found are historically considered to function either in metabolism and longevity *or* in immunity. However, we highlight that these pathways not only cross-talk, but also clearly act pleiotropically to regulate pathogen resistance, lifespan, and ageing among many other physiological processes such as metabolism and stress resistance.

We also identified several cases where the effects of these pathways were not consistent, but varied between tissues, experimental conditions, sexes and pathogens. Classifications of genes into ‘pro’ and ‘anti’ longevity/immunity might therefore present a wrong or incomplete picture in many cases as context-dependency needs to be considered. Irrespective of that, we attempted to generalize the effect of each discussed pathway on lifespan and immunity (see Table 1) to discover trade-offs and functional conservation. Based on Table 1, we hypothesize that IIS/TOR, RNAi, and Nf-κB tend to be more functionally conserved than other pathways. Among these pathways, only the Nf-κB pathway clearly modulates a lifespan-immunity trade-off, as its activation facilitates pathogen defence but reduces lifespan. A good candidate mechanism explaining this is that triggering the Nf-κB cascades drives inflammatory and antimicrobial processes, which are detrimental for pathogens and the infected host at the same time. In contrast, activation of IIS/TOR mostly promotes growth in the presence of nutrients at the cost of *both* immunity and lifespan. The reallocation of resources between these phenotypes is mediated by Foxo transcription factors that transcribe longevity-conferring genes but concurrently upregulate immunity and stress-response genes. Finally, the RNAi pathway, which is also influenced by the IIS/TOR network, generally facilitates longevity and pathogen resistance, that could be resulting from regulation of gene expression and silencing of transposable elements in addition to its antiviral function.

**Table 1:**
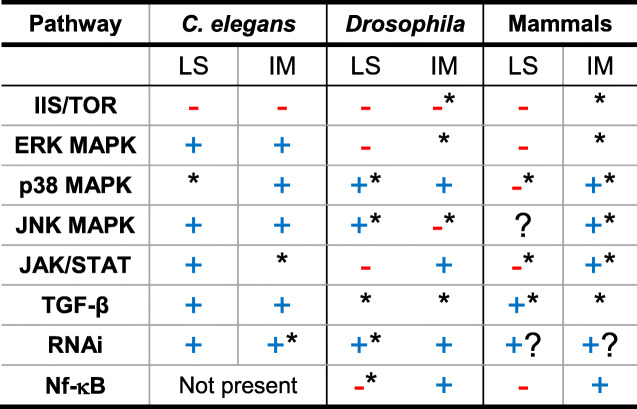
Simplified overview of functional conservation in immuno-ageing pathways between *C. elegans*, *D. melanogaster*, and mammals. The table outlines a general simplification of how pathway activation affects lifespan (LS) or immunity (IM). Positive effects of pathway activation, such as improved lifespan or immunity, are indicated by a blue “+”, negative with “- “in red. A star (*) symbol signifies that the effects are to some degree context-dependent (e.g. vary between tissues or sexes). The question mark (?) shows that the relationship between pathway and lifespan/immunity phenotype has not been studied or is too poorly understood to identify a general pattern

Notably, most of the immuno-ageing pathways were identified through loss-of-function and gene knockdown assays, while insights from studies on population-wide genetic variation are limited. Most evidence for how alleles in these pathways affect ageing and immunity comes from *Drosophila* and humans. For instance, variants in Nf-κB related immunity genes were associated with longevity and improved immunosenescence in *Drosophila* [195]. In humans, FOXO3 and the cytokine interleukin 6 (IL6), which induces JAK/STAT, ERK-MAPK and PI3K signalling, are two prominent examples for which genetic variability has been linked with variation in lifespan and inflammation [98, 215–217]. This highlights that methods connecting genomic and phenotypic variation, such as genome-wide association studies, are powerful tools that will be further utilized to understand immuno-ageing genes and pathways.

Our review demonstrates that loss of immune homeostasis is a central determinant of ageing across diverse phyla. Yet, whether immunosenescence and the age-associated decline in other traits is a cause or result of ageing remains a fundamental problem difficult to resolve. Knowing the exact time and place of changes related to immunity and ageing would be a huge step in answering this question. Moreover, how environmental effects, including life-long pathogenic challenges, variation in the microbiome, or nutrition affect age-related changes in immunity is poorly understood. To date most studies are restricted in resolution, particularly in terms of analysed tissues, time points, phenotypes and experimental conditions. Cutting-edge technologies such as single-cell sequencing can be useful in that respect and could be utilized to characterize molecular changes during ageing and infection in specific cell types. In combination with genome-wide CRISPR knockout screens, new immuno-ageing genes can be discovered and the cross-talk between immunity and ageing further deciphered. Currently, the level of detail needed to solve the causality enigma of ageing is likely not achievable in humans but may be addressed in shorter lived model organisms that are easier to manipulate. Once we understood ageing at this unprecedented level, it will be possible to optimize lifestyle factors and emerging drug therapies treating senescence to facilitate healthy ageing and extend lifespan.

## Supplementary Information

**Additional file 1: Table S1.** Immuno-ageing, longevity/ageing, and immunity genes. To define immuno-ageing genes (‘ImmAg’ sheets) we assembled all currently known immunity and longevity/ageing genes (‘Imm’ and ‘Ag’ sheets) from various databases and identified genes occurring in both traits (see ‘Summary’ tab). This was done for two invertebrates, *C. elegans* and *D. melanogaster* (‘Cele’ and ‘Dmel’ sheets), and two mammals, *M. musculus* and *H. sapiens* (‘Mouse’ and ‘Human’ sheets). Ageing genes were obtained from the GO term ‘aging’ (GO:0007568), the KEGG pathway annotation ‘Longevity regulating pathway’ (KEGG id: 04213), and two ageing databases GenAge and AgeFactDB. Immunity genes were compiled from the GO term ‘immune system process‘ (GO:0002376), multiple immune-related KEGG pathways (for *D. melanogaster* only available KEGG id: 04624; while for mammals we used: 04640, 04610, 04611, 04620, 04621, 04622, 04623, 04625, 04650, 04612, 04660, 04658, 04659, 04657, 04662, 04664, 04666, 04670, 04672, and 04062), and the three immunity databases insect innate immunity database IIIDB, InnateDB, and immunome knowledge base (IKB). Genes from GO and KEGG were obtained using the biomaRt package in R, while genes from databases were downloaded from the corresponding websites. We added additional annotations (such as IDs from multiple databases) using biomaRt, and the species-specific browsers WormBase for *C. elegans*, FlyBase for *D. melanogaster*, MGI for *M. musculus*, and HGNC for *H. sapiens*. For AgeFactDB, we excluded all genes for which the experimental evidence was annotated as ‘putative’ or ‘no’. Annotations were obtained in January 2019 for IIIDB and Immunome, and August 2020 for all others. Additional genes with clear effects on immunity and ageing, which we found reviewing the literature, were manually added for *C. elegans* and *D. melanogaster* (‘handcurated’ column; references below). The number of genes obtained from each database are given in the ‘Summary’ sheet. The column ‘Longevity’ in the gene lists indicates whether a gene is beneficial (pro) or detrimental (anti) for lifespan or had no effect (none) as defined by the GenAge database.

**Additional file 2: Table S2.** Shared immuno-ageing orthologs. The table gives details to the 437 human orthologs/genes in the partitions of the cross-species VennDiagram in Fig. 2. Human orthologs of immuno-ageing genes of all species were obtained using DIOPT (Option: Return only best match when there is more than one match per input gene or protein). The species and count of species sharing a human immuno-ageing ortholog are given in the column “Overlap” and “SpeciesCount”, respectively.

## Data Availability

All assembled gene lists are available in Table S1 and Table S2.

## Abbreviations

AMP: Antimicrobial peptides
EGFR: Epidermal growth factor receptor
ERK: Extracellular-signal-regulated kinase
GO: Gene Ontology
IFN: Interferon
IGF: Insulin-like growth factor
IIS: Insulin-insulin-like growth factor signalling
JAK/STAT: Janus kinase / signal transducer and activator of transcription
JNK: c-Jun N-terminal kinase
KEGG: Kyoto Encyclopedia of Genes and Genomes
MAPK: Mitogen-activated protein kinase
MHC: Major histocompatibility complex
mTORC1/2: Mammalian target of rapamycin complex 1/2
Nf-κB: Nuclear factor kappa-light-chain-enhancer of activated B cells
PI3K: Phosphatidyl-inositol 3 kinase
PGRP: Peptidoglycan recognition proteins
PRR: Pattern recognition receptors
RNAi: RNA interference
ROS: Reactive oxygen species
TGF-β: Transforming growth factor beta
TOR: Target of rapamycin
